# Study protocol for a pragmatic randomized controlled trial evaluating coarse-cereal intake intervention for obesity prevention among primary school students in suburban Beijing, China

**DOI:** 10.3389/fpubh.2026.1812397

**Published:** 2026-04-14

**Authors:** Qingbin Xing, Juan Xu, Titi Yang, Hongliang Wang, Jianfen Zhang, Ruihe Luo, Wei Cao, Hui Pan, Xuelian Guo, Jun Zhang, Le Yan, Xue Yan, Qi Su, Zhu Wang, Qian Zhang

**Affiliations:** 1 Key Laboratory of Public Nutrition and Health, National Institute of Nutrition and Health, Chinese Center for Disease Control and Prevention, Beijing, China; 2 Beijing No. 2 Experimental Primary School Huairou Branch, Beijing, China; 3 Beijing Zhongguancun No. 1 Primary School Huairou Branch, Beijing, China; 4 Huairou Center for Disease Control and Prevention, Beijing, China; 5 Primary and Secondary School Health Care Center of Huairou District, Beijing, China

**Keywords:** children, coarse-cereal, Custer randomized trial, dietary Fiber, obesity

## Abstract

**Background:**

The rising prevalence of childhood obesity poses a significant public health challenge in China, with rates among primary school students increasing from 6.8% in 2002 to 19% in 2020. Concurrently, coarse-cereal intake—a source of dietary fiber and bioactive compounds with potential protective effects against obesity—has declined markedly. School-based interventions represent a feasible strategy to address this issue. This protocol describes a cluster-randomized pragmatic controlled trial (pRCT) aimed at evaluating the effectiveness of a comprehensive intervention to increase coarse-cereal intake for obesity prevention among Chinese primary school students.

**Methods:**

A two-arm pragmatic cluster-RCT will be conducted in public primary schools in Huairou District, Beijing. Two schools will be randomly assigned to intervention or control groups, with 400 students (grades 3–4, aged 8–12 years) enrolled. The intervention group will receive a daily 50 g coarse-cereal substitution in school meals, aligned with Chinese dietary guidelines, alongside a multi-level nutrition education program targeting students, parents, and school staff. The control group will continue standard practices. Primary outcomes include changes in body mass index standard deviation score (BMI-SDS), body composition, waist circumference, and blood pressure, assessed at baseline, 3 months, and 6 months. Secondary outcomes encompass metabolic indicators and gut microbiota profiles in overweight/obese subgroups. Data will be analyzed using mixed-effects linear models, accounting for cluster effects. Implementation fidelity will be monitored through quantitative and qualitative methods.

**Discussion:**

This trial will address a critical evidence gap by testing a culturally adapted, school-based intervention to promote coarse-cereal consumption. If effective, the findings will inform the integration of coarse cereals into national student meal policies, contributing to sustainable obesity prevention strategies in China. The study design emphasizes practical implementation through a socio-ecological framework, with potential for scalability.

**Clinical trial registration:**

Identifier ChiCTR2500114773.

## 1 Introduction

The prevalence of overweight and obesity among children and adolescents has increased substantially worldwide. According to the World Obesity Federation, the combined prevalence of overweight and obesity among individuals aged 5–19 years rose from approximately 8% in 1990 to nearly 20% in 2022 (1). In 2022, more than 390 million children and adolescents in this age group will be classified as overweight, including approximately 160 million who will be obese. A similar trend has been observed in China, where the prevalence of the overweight and obesity among primary school students increased from 6.8% in 2002 to 19% in 2020 (2, 3). This increase has occurred not only in urban areas but also in rural regions (3). Childhood obesity is associated with an elevated risk of adverse health outcomes later in life, including type 2 diabetes, cardiovascular diseases, and metabolic syndrome, thereby increasing the long-term economic burden and placing substantial pressure on healthcare systems (4, 5).

Dietary factors play a critical role in the development of obesity, and cereals constitute a major component of the traditional Chinese diet (6). Cereals are commonly classified as refined cereals or coarse cereals. Refined cereals undergo extensive processing, resulting in substantial losses of vitamins, minerals, and dietary fiber, with starch remaining the predominant component. High consumption of refined cereals has been associated with an increased risk of overweight and obesity (7–10). In contrast, coarse cereals are minimally processed or unprocessed and share characteristics with whole grains, retaining higher levels of dietary fiber and various bioactive compounds (9). Historically, coarse cereals represented a major staple in the Chinese diet. However, their consumption has declined markedly over recent decades. In 1982, Chinese children aged 6–17 years consumed an average of 114.3 g of coarse cereals per day, whereas intake decreased to only 8.0 g per day by 2020 (3, 11).

Several studies have suggested that increasing the intake of fiber-rich cereal foods may be beneficial for the prevention and control of overweight and obesity among school-aged children (12–16). For example, a school-based whole-grain provision program combined with parental education in Malaysia was associated with reductions in BMI z-scores and body fat percentage over a six-month intervention period; compared with the control group, children in the intervention group experienced a 2.6% reduction in body fat percentage and a 2.4 cm decrease in waist circumference (17). Similarly, the Healthy School Meals Initiative in the United States has been shown to improve dietary quality and was associated with lower prevalence rates of overweight and obesity in participating schools (18).

Taken together, the increasing prevalence of childhood obesity in China, the marked decline in coarse-cereal consumption, and the potential of schools as effective intervention settings underscore the importance of the present study. By evaluating the effectiveness of a school-based coarse-cereal intake intervention, this study aims to address existing evidence gaps and generate evidence to inform strategies for childhood obesity prevention in China.

## 2 Methods and analysis

This study protocol is reported in accordance with the Standard Protocol Items: Recommendations for Interventional Trials (SPIRIT) 2025 guidelines.

### 2.1 Study design

**Figure 1:**
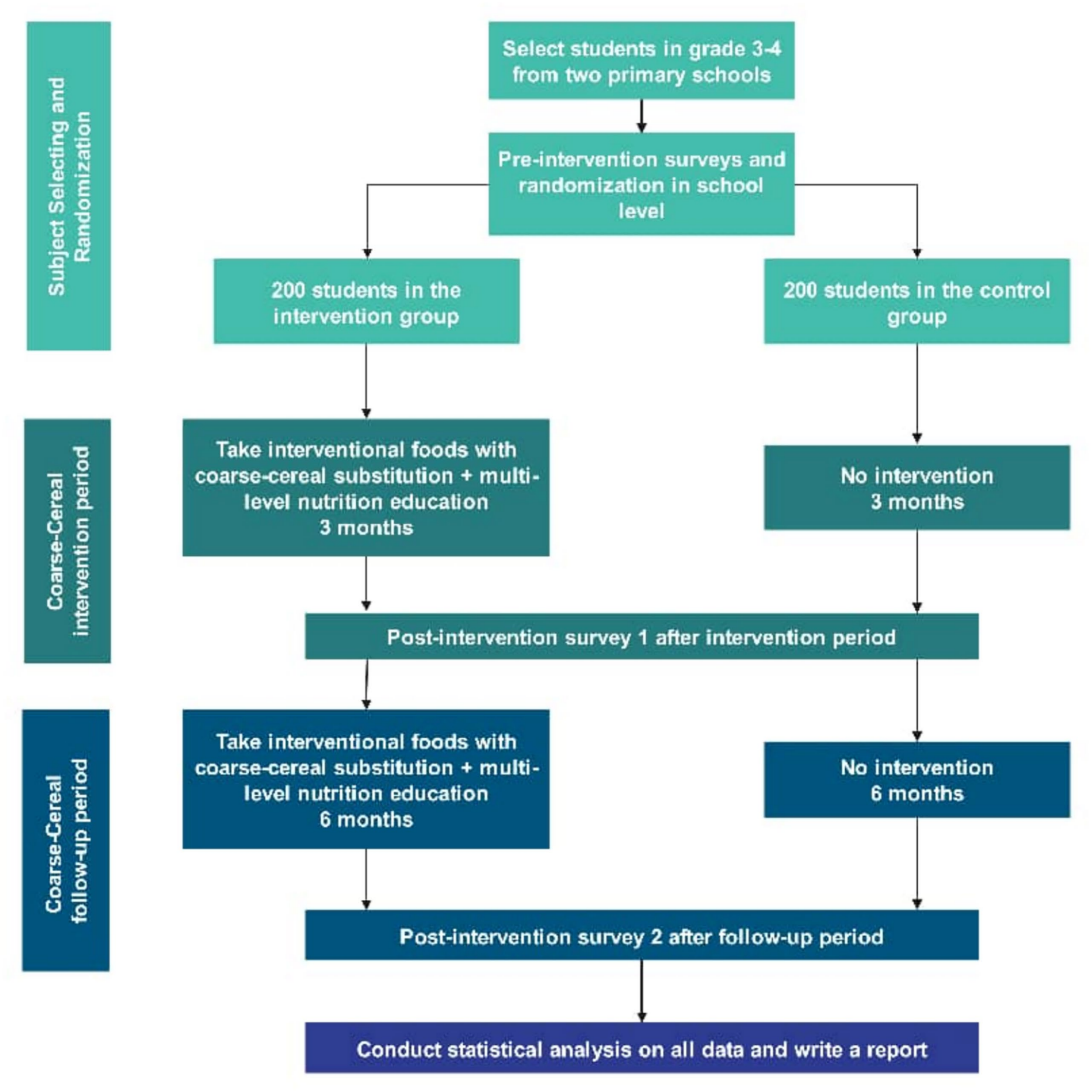
Flowchart of intervention process.

This study is designed as a parallel-group, cluster-randomized pragmatic controlled trial (pRCT) with two arms: an intervention group receiving a coarse-cereal intake intervention and a control group continuing usual dietary practices (19). Recognizing the real-world constraints of school-based dietary interventions, we adopted a pragmatic design to evaluate the effectiveness of the intervention under routine conditions. Consequently, while blinding of participating students, parents, and school staff is inherently unfeasible due to the nature of the dietary intervention, this limitation is an accepted characteristic of the pRCT framework aimed at maximizing external validity. To mitigate potential assessment bias within this pragmatic framework, outcome assessors responsible for anthropometric and biological measurements will remain blinded to group allocation. The study protocol adheres to the CONSORT 2025 extension for pragmatic trials to ensure methodological rigor and transparency (20, 21). The study commenced in March 2026, and the basic process of the intervention is illustrated in Figure 1.

### 2.2 Randomization and allocation

Clusters, defined as individual schools, serve as the unit of randomization. The study population is drawn from public primary schools in Huairou District, Beijing, where the prevalence of overweight and obesity among school-aged children exceeds 30%, highlighting the need for effective interventions.

Eligible schools met the following criteria: (1) Availability of an on-site cafeteria capable of providing daily breakfast and lunch to ensure consistent implementation of coarse-cereal substitution; (2) No dietary intervention programs implemented in the past year; (3) Enrollment of at least 150 students in grades 3–4 (ages 8–12) to ensure sufficient sample size per cluster, and (4) Approval from school administrators to participate in the trial.

Two schools meeting these criteria will be randomly assigned to either the intervention or control group using a computer-generated random number table (SAS 9.4).

### 2.3 Study population

All students in grades 3–4 at the selected schools will be screened for individual eligibility according to the inclusion and exclusion criteria. A total of 400 students will be enrolled, with 200 allocated to the intervention group and 200 to the control group, allowing for potential attrition.

#### 2.3.1 Sample size calculation

The primary outcome for sample size estimation was the Body Mass Index Standard Deviation Score (BMI-SDS). Due to the lack of previous studies on coarse-cereal interventions in this population, sample size was estimated based on prior research evaluating comprehensive interventions on obesity in primary school students. The calculations assumed 80% power (*β* = 0.20), a two-sided significance level of 0.05 (*α* = 0.05), standard deviation *σ* = 1.39, and effect size *Δ* 0.17. The resulting sample size was 180 students per school. Allowing for 10–20% attrition, the target enrollment per school was set at 190–200 students (18). The calculation formula used will be:


$$n=\frac{{\left(Z_{\alpha /2}+Z_{\beta }\right)}^{2}{\left(2\sigma^{2}\right)}^{2}[1+(m-1)\rho ]}{{\left(\mu_{1}-\mu_{2}\right)}^{2}}$$

#### 2.3.2 Inclusion criteria

Children enrolled in grades 3–4 in the specified region will be eligible provided that written informed consent is obtained from a parent or guardian. To ensure safety and feasibility, participants must be in good general health, excluding those with severe acute or chronic conditions that would prevent full participation in standard school activities.

#### 2.3.3 Exclusion criteria

Students will be excluded if they: (1) Had diagnosed allergies or intolerances to coarse cereals, and (2) Had chronic diseases confirmed by a secondary hospital or higher (eg. type 1 diabetes, congenital heart disease) that could affect dietary intake, metabolism or growth.

### 2.4 Intervention strategies

#### 2.4.1 Intervention group

Eligible students aged 8–12 years (grades 3–4) in the intervention school received daily coarse-cereal intervention incorporated into school meals, combined with structured nutrition education for students and parents.

#### 2.4.2 Coarse-cereal substitution in school meals

##### 2.4.2.1 Coarse-cereal substitution

According to the Dietary Guidelines for Chinese Residents (22), children aged 7 ~ 13 years should consume 30–70 g of coarse cereals or legumes daily. In the intervention school, approximately 50 g of coarse cereals per student per day will be provided through the substitution of refined grains in school meals. This target amount is within the recommended range and will be integrated into breakfast, lunch, and dinner (the latter for boarding students only).

To ensure dietary diversity and palatability, the research team, in collaboration with school kitchen staff, developed a standardized coarse-cereal menu. The intervention will utilize common types of coarse cereals and legumes such as oats, brown rice, buckwheat, millet, corn grits, red beans, and mung beans. Over 100 dishes across 12 categories (e.g., buckwheat noodles, red bean rice, millet congee, oat steamed bread) were created, with menus designed to rotate weekly. The core substitution method is a direct, gram-for-gram replacement: coarse cereals will constitute a portion of the staple food, for instance, by using brown rice to partially or wholly replace white rice, or by incorporating whole wheat/oat flour into wheat flour for making buns and noodles. Each daily menu is designed to provide approximately 50 g of coarse cereals and about 4 g of dietary fiber per student, based on calculations from the China Food Composition Tables.

A registered dietitian will audit the school meal menus every 2–3 days to verify coarse-cereal types, portion sizes, and rotation frequency. Coarse cereals will be prepared by steaming, boiling, or stewing to minimize nutrient loss. Recipes were adjusted after pilot testing to balance nutrient content and student acceptance to optimize compliance.

#### 2.4.3 Multi-level nutrition education

##### 2.4.3.1 Student education

Students will attend 7 structured, interactive nutrition education sessions (approximately 45 min each, conducted twice per month). These sessions will be delivered by trained school health teachers or homeroom teachers using a standardized curriculum developed by the research team. The curriculum covers sequential topics including: (i) Introduction to Coarse Cereals and Their Nutritional Value; (ii) The Food Guide Pagoda and Building a Balanced Diet; (iii) Understanding Obesity and Its Health Risks; (iv) Making Healthy Snack and Beverage Choices; and (v) Practical Activities (e.g., comparing brown and white rice, designing a healthy lunch box). To ensure consistency and quality, teachers will receive a two-hour training workshop and a detailed lesson plan kit prior to the intervention. Research assistants will conduct random spot checks (via classroom observation or lesson recording review) to monitor fidelity to the protocol.

##### 2.4.3.2 Parent engagement

Parents or guardians will receive nutrition education through two WeChat messages per month (totaling six sessions) sent via class group chats. The messages will cover practical topics such as home cooking methods for coarse cereals, interpreting nutrition labels, and preparing healthy after-school snacks. Additionally, four live webinars focusing on child nutrition and weight management will be hosted by project nutritionists and scheduled on weekday evenings to maximize accessibility. To encourage home environment support, school principals and teachers will also receive summarized briefings on the key messages. Parent engagement will be monitored through metrics such as message read-receipt rates and webinar attendance logs.

##### 2.4.3.3 Control group consideration

To maintain ethical rigor, the control group will not receive any active nutrition education during the 6-month intervention period. Upon study completion, they will be provided with access to the compiled educational materials (e.g., lesson recordings, digital resources).

#### 2.4.4 Control group

Students in the control schools will continue to receive standard school meals without coarse-cereal substitution and will not receive any nutrition education during the intervention and follow-up periods. To ensure ethical rigor, the control group will be provided with access to the same educational materials (including lesson recordings, lesson plans, and digital resources) upon completion of the 6-month follow-up.

### 2.5 Data collection

**Figure 2:**
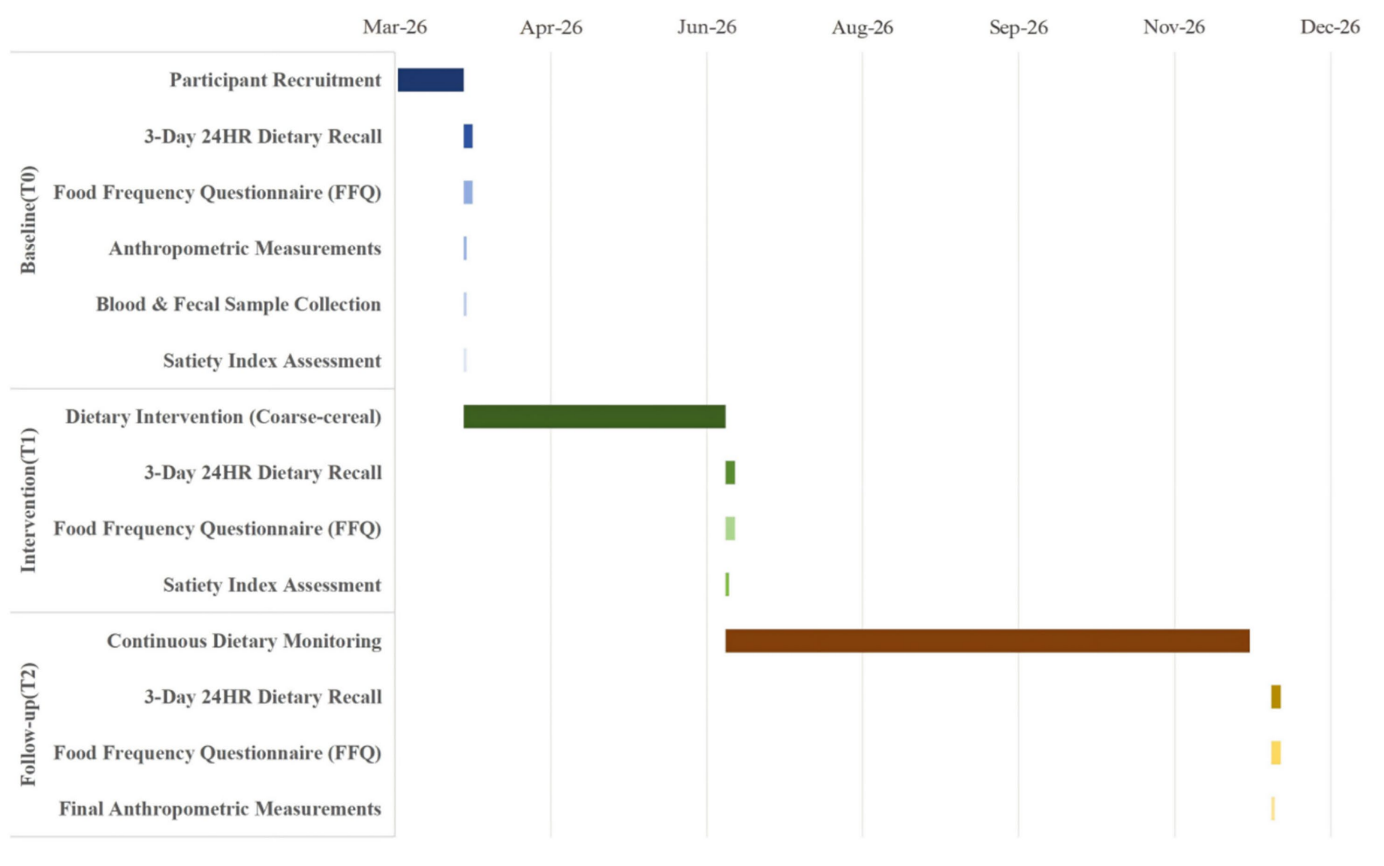
Data collection timeline for coarse-cereal intervention study.

Data will be collected at three time points: baseline (T0), post-intervention at 3 months (T1), and at 6-month follow-up (T2, 9 months). The specific time points for each category of data collection are detailed in Figure 2.

#### 2.5.1 Exposure indicators

These indicators will quantify actual coarse-cereal intake in the intervention group, assess adherence to the 3-month intervention, and compare intake levels with the control group’s usual diet.

##### 2.5.1.1 3-Day 24-hour dietary recall (3D-24HR)

Students recorded all food and beverage intake over three consecutive days (two weekdays, one weekend) at T0, T1, and T2. Kitchen scales will be provided to measure portions, and cooking methods will be recorded. Canteen staff documented daily menus to calculate in-school coarse-cereal intake. Parents maintained weekly coarse-cereal purchase and cooking logs. Daily intake (g/day) will be calculated using the China Food Composition Tables.

##### 2.5.1.2 Food frequency questionnaire (FFQ)

A validated, child-specific FFQ (23) will assess habitual diet over the past month at baseline (T0), post-intervention at 3 months (T1), and at 6-month follow-up (T2, 9 months), including 65 items across 11 food categories. The frequency and portion sizes of coarse cereals, sugar-sweetened beverages, and snacks will be recorded.

#### 2.5.2 Outcome indicators

Primary outcomes will be selected to assess the direct effect of increased coarse-cereal intake on overweight and obesity. Secondary outcomes, including indicators related to metabolism and gut microbiota, will be measured at baseline (T0) and at the end of the intervention (T1, 3 months).

##### 2.5.2.1 Primary outcome indicators

(1) Body Mass Index (BMI)

Height and weight will be measured using a calibrate stadiometer and digital scale, with students in light clothing and barefoot after fasting. BMI will be calculated as weight (kg)/ height^2^ (m^2^) and categorized according to Chinese reference standards.

(2) Body Composition

Measured using an 8-electrode, 4-frequency bioelectrical impedance analyzer (InBody 770, Biospace Co., Ltd.) validated for children to assess body fat and lean mass.

(3) Waist Circumference (WC)

Measured at the midpoint between the lowest rib and iliac crest with a non-elastic tape, in triplicate, to 0.1 cm accuracy.

(4) Blood Pressure (BP)

Measured using an electronic sphygmomanometer (HBP-1300, Omron) after 5 min of rest; two readings will be taken with a 3-min interval. Childhood obesity is a significant risk factor for elevated blood pressure, which serves as a sentinel indicator for assessing cardiometabolic risk. By incorporating blood pressure measurements, this study aims to investigate whether increasing dietary fiber intake through coarse-cereal consumption offers potential benefits in improving early cardiovascular risk markers associated with obesity.

##### 2.5.2.2 Secondary outcome indicators

(1) Metabolic Indicators

After an 8–10 h overnight fast, 5 mL of venous blood will be collected from 200 overweight/ obese students. Serum will be centrifuged and stored at −80 °C.

(2) Carbohydrate metabolism

Fasting blood glucose (FBG), fasting insulin (FI), and insulin-like growth factor 1 (IGF-1) will be assessed.

(3) Lipid metabolism

Lipid profile (enzymatic colorimetric method) and adipokines (leptin, leptin receptor, adiponectin via ELISA) will be measured. Serum uric acid (SUA) will be also assessed.

(4) Gut Microbiota

Forty overweight/ obese students per group provided ~5 g fecal samples at T0 and T1, stored at −80 °C. 16S rRNA V4-V5 sequencing will be performed to assess alpha diversity (Shannon index), beta diversity (Bray-Curtis distance), and relative abundance of fiber-degrading bacteria (e.g., *Bifidobacterium, Akkermansia*).

#### 2.5.3 Dietary behavior-associated indicators

Satiety index (SI) will be assessed on 2 working days via a 10-point scale after breakfast and lunch.

#### 2.5.4 Influencing indicators

Potential confounders will be measured and adjusted in analysis:

(1) Physical Activity and Sedentary Behavior

Students will wear smart wristbands for 7 consecutive days (including 5 school days and 2 weekend days) at baseline (T0) and at the end of the 3-month intervention (T1) to measure activity duration and frequency, sedentary time, and sleep.

(2) Dietary Preference and Cognition

Dietary preferences, knowledge, and home environment will be assessed using a structured questionnaire developed for this study. The questionnaire items were generated based on a literature review and expert consultation (involving pediatric nutritionists and epidemiologists), and its face validity was confirmed through pilot testing with a small group of non-participant students and parents for clarity and comprehension. It assessed preference for coarse cereals, ability to identify coarse-cereal foods, snacks choices, parental home coarse-cereal availability, cooking frequency, and role modeling (23).

(3) Baseline Demographic

Age, sex, family information, and other health data.

### 2.6 Statistical analysis

Analysis accounts for the cluster-randomization design, with schools as clusters and students nested within schools. Intention-to-treat (ITT) principles are applied, using SAS 9.4.

#### 2.6.1 Descriptive statistics and baseline comparison

Continuous variables are presented as mean±standard deviation (SD) or median (skewed). Normality will be tested via Shapiro–Wilk test and Q-Q plots. Categorical variables are summarized as counts (%). Baseline differences will be assessed using t-tests or Mann–Whitney U tests for continuous variables, and χ^2^or Fisher’s exact tests for categorical variables.

#### 2.6.2 Outcome analysis

Analysis will be performed following the intention-to-treat principle. Mixed-effects linear models (MLMs) will be used to evaluate changes in outcomes from baseline (T0) to post-intervention (T1) and follow-up (T2). The models will include group (intervention/control), time (T1, T2), and the group-by-time interaction as fixed effects, with the baseline value (T0) of the respective outcome included as a covariate. Individual-level intercepts will be fitted as random effects to account for repeated measurements, and school will be included as a random effect to adjust for the cluster-randomized design. The models will be further adjusted for prespecified covariates, including age, sex, and baseline physical activity level. The primary test for intervention effect will be the significance of the group-by-time interaction term.

#### 2.6.3 Subgroup and sensitivity analyses

Pre-specified subgroups included se and baseline BMI category. Three way interactions (group x time x subgroup) tested effect modification, with Bonferroni correction (*α* = 0.025). Per-protocol analysis excluded participants with <50% intervention adherence. Complete-case analysis will be conducted to assess robustness to missing data.

#### 2.6.4 Quality assurance and control procedures

To ensure data quality, the following measures will be implemented: (1) All data collectors will receive standardized training and certification. (2) Measurement equipment will be calibrated regularly. (3) Key anthropometric measurements (height, weight, waist circumference) will be taken in duplicate by independent staff; a third measurement will be taken if the difference exceeds pre-set tolerances, with the median value recorded. (4) Strict Standard Operating Procedures (SOPs) will be followed for the collection, processing, and storage of all biological samples. (5) Data will be entered electronically using a double-entry system with validation checks to ensure accuracy.

### 2.7 Implementation evaluation

Implementation of fidelity and exposure will be assessed to validate intervention effects. Metrics will include:

(1) School-level compliance with the meal plan (e.g., provision of ≥50 g of coarse cereals per student per day).
(2) Participant attrition rates at T1 and T2.
(3) Comparison of overweight/obesity outcomes between compliant and non-compliant schools.
(4) Individual-level adherence, which will be assessed based on the 3-day 24-h dietary recall data collected at T1. High adherence is defined as an estimated average daily coarse-cereal intake of ≥40 g, while low adherence is defined as an intake of <25 g.
(5) A prespecified subgroup analysis comparing outcomes between participants with high adherence and those with low adherence.

Paired *t*-tests and standardized regression coefficients will be used to evaluate outcome changes and the impact of confounding factors. Analyses will adjust for confounding factors (e.g., age, sex, baseline BMI). The impact intensity of each variable will be determined using standardized regression coefficients.

## 3 Discussion

### 3.1 Rationale and significance

Coarse cereals, including millet, naked oats, and buckwheat, have a long history in the Chinese diet (6). Despite dietary guidelines recommending 30–70 g of coarse cereals per day for children aged 7–13 years (22), average intake among Chinese children was 8 g/day in 2020. Concurrently, school meal programs in China are being standardized and widely implemented, providing a feasible platform to improve dietary quality and reach a large proportion of school-aged children (24, 25). By integrating coarse-cereal provision into daily meals, this intervention is culturally acceptable and likely to be supported by parents and schools.

This study protocol outlines a school-based pragmatic randomized controlled trial (pRCT) designed to evaluate the real-world effectiveness of a daily 50 g coarse-cereal substitution in preventing obesity among primary school students (grades 3–4, aged 8–12 years) (19). Unlike explanatory trials conducted under highly controlled conditions, this pRCT leverages existing school infrastructure and routine operational workflows to assess the intervention’s impact within the complex reality of the Chinese educational setting. The intervention combines dietary modification, multi-level nutrition education, and digital monitoring tools, addressing critical gaps in childhood obesity prevention research in China. By adopting a pragmatic design, this study aims to generate robust evidence on the feasibility, scalability, and practical effectiveness of coarse-cereal interventions, thereby directly aligning with national public health priorities and informing future policy implementation.

### 3.2 Theoretical framework and innovation

The intervention is grounded in an ecological perspective of dietary behavior (8, 26) and employs culturally and environmentally adapted strategies. Locally familiar foods (e.g., brown rice, oats, red beans) are used, and over 100 standardized recipes are tailored to school kitchen capacities. This approach addresses low childhood intake of coarse cereals and ensures intervention sustainability (27), filling a gap in global research where environmental feasibility is often overlooked.

Bronfenbrenner’s Social-Ecological Model (SEM), recognized by the WHO as a framework for health interventions (28), informs the multi-level design, linking schools and families to enhance behavioral change (29). Biological mechanisms are also incorporated through gut microbiota analysis (16S rRNA sequencing of fecal samples) and metabolic biomarkers, providing mechanistic insight into how dietary interventions may affect obesity outcomes.

### 3.3 Future directions and practical applications

To evaluate long-term effects, future studies should extend intervention and follow-up periods to 12 months, enabling assessment of sustained coarse-cereal consumption and obesity prevention. Assessing the feasibility of independent school-based provision after the intervention is critical. Adaptation for rural schools, where on-site cafeterias are often absent, may involve pre-packaged coarse-cereal snacks or parent-led meal preparation while maintaining the 50 g daily target. These strategies could reduce rural–urban disparities in childhood obesity and align with China’s Rural Compulsory Education Student Nutrition Improvement Program.

Subgroup analyses by gender and baseline weight status could refine intervention targeting, focusing on high-risk populations if differential efficacy is observed. Linking gut microbiota changes to metabolic outcomes, such as insulin sensitivity and lipid profiles, may support development of microbiome-informed, personalized interventions.

If proven effective, the findings and resources from this study are intended to inform practical applications and policy. The standardized resources developed, including the recipe library, digital monitoring tools, and staff training protocols, could be consolidated into a “Coarse-Cereal School Toolkit” for broader adoption. The toolkit would provide a tangible pathway for integrating coarse cereals into national policies, such as an update to the Nutritional Guidelines for Student Meals (24), potentially mandating or strongly recommending the inclusion of coarse cereals in school meal programs.

However, scaling up such an intervention nationwide would face several implementation challenges. These may include: variability in local food supply chains and kitchen capacities, the need for additional staff training, potential higher upfront food costs, and ensuring consistent student acceptance across diverse regions. To overcome these challenges, phased implementation strategies could be considered, starting with pilot regions. Other supportive strategies might include: establishing procurement guidelines and partnerships with local producers to ensure cost-effectiveness and supply; developing tiered training modules adaptable to different school settings; and conducting ongoing sensory testing and menu optimization to maintain palatability. By proactively addressing these potential barriers, the transition from research evidence to sustainable, large-scale practice can be facilitated.

### 3.4 Limitations

Several limitations should be considered within the context of this pragmatic design. First, the 3-month intervention period may not capture long-term effects, although the 6-month follow-up provides a partial evaluation of maintenance. Second, as a pilot pragmatic cluster-randomized trial (pRCT) conducted in only two primary schools in suburban Beijing, the findings are primarily applicable to similar settings with on-site cafeterias. While the limited number of clusters may increase the risk of baseline imbalance, this pilot scale was necessary to test the feasibility and implementation fidelity of the intervention before wider rollout. Third, consistent with the nature of pragmatic trials conducted in real-world settings, blinding of participants and school staff was not feasible. Although this introduces the potential for performance bias, it reflects the actual conditions under which such dietary interventions would be implemented. To mitigate detection bias, we strictly maintained blinding of outcome assessors for anthropometric and biological measurements. Finally, unmeasured confounders, such as family lifestyle factors not captured in current assessments, may influence outcomes, a challenge common to school-based community interventions.

## 4 Conclusion

This school-based pragmatic randomized controlled trial (pRCT) addresses a critical public health need by evaluating a contextually appropriate coarse-cereal intervention to prevent childhood obesity in China. By emphasizing cultural relevance, multi-level engagement, and real-world applicability, the study is expected to provide actionable evidence on both the effectiveness and feasibility of updating national student meal policies. While explanatory trials establish efficacy under ideal conditions, this pRCT is designed to inform how such interventions perform within the complex constraints of routine school environments. Despite inherent limitations, the protocol’s rigorous design—including cluster randomization, blinded outcome assessment, and comprehensive quality control—ensures that results will be robust and directly translatable to policy, ultimately contributing to the health of Chinese school-aged children.

## Data management and confidentiality

To protect participant privacy, all collected data will be de-identified. Each participant will be assigned a unique identification code. The key linking codes to personal identifiers will be encrypted and stored separately, accessible only to the principal investigator and designated data managers. Paper records containing personal information will be stored in locked cabinets. Electronic data will be stored on password-protected servers behind institutional firewalls. All research team members are required to sign confidentiality agreements. Data will be retained for a minimum of 5 years after study completion and will be used solely for the pre-specified analyses in this protocol or for future secondary analyses (subject to separate approval by the ethics committee).
